# Schizotypy and subclinical depression relate to specific trait curiosity dimensions

**DOI:** 10.1038/s41598-026-61554-y

**Published:** 2026-07-17

**Authors:** Heike Sönnichsen, Alexandra Sobczak, Tineke K. Steiger, Nico Bunzeck

**Affiliations:** 1 Department of Psychology, University of Luebeck, Maria-Goeppert-Strasse 9a, 23562 Luebeck, Germany; 2 Center of Brain, Behavior and Metabolism (CBBM), University of Luebeck, Luebeck, Germany

**Keywords:** Curiosity, State, Trait, Depression, Schizotypy, Diseases, Neuroscience, Psychology, Psychology

## Abstract

Curiosity promotes human development, but its link to subclinical psychological variations remains poorly understood. In two online experiments, we examined associations of trait and state curiosity with schizotypal characteristics (experiment 1, *n* = 238) and subclinical depressive symptoms (experiment 2, *n* = 264) in samples from the general population. In addition to an established multidimensional curiosity framework that allowed us to quantify specific trait curiosity facets, such as Joyous Exploration, Stress Tolerance and Deprivation Sensitivity, we also employed a previously established test to assess state epistemic curiosity. Both schizotypal characteristics and subclinical depressive symptoms were associated with lower Joyous Exploration and Stress Tolerance, while higher Deprivation Sensitivity showed more selective associations with schizotypy. In contrast, state epistemic curiosity did not significantly correlate with schizotypy or depressive symptoms. Together, these findings suggest that trait curiosity dimensions show both shared and selective associations with subclinical depression and schizotypy, potentially reflecting distinct motivational and affective processes. From a broader perspective, this underscores the value of a multidimensional approach to curiosity in mental health.

**Supplementary Information:**

The online version contains supplementary material available at 10.1038/s41598-026-61554-y.

## Introduction

From early childhood on, curiosity plays a critical role in learning, cognitive development, and adaptive behavior, and has, therefore, been examined in relation to educational achievement, personality, or motivation^1,2^. Growing evidence further suggests that curiosity is also intertwined with mental health across the human lifespan, indicating its potential as a protective factor and marker of vulnerability. While often portrayed as a positive and growth-oriented trait, curiosity can vary considerably across individuals and may be shaped, or even impaired, by subclinical or clinical psychological conditions. Evidence in favor of such a view, however, is limited. Therefore, this work aims to further understand the concept of curiosity in the context of two subclinical traits with high psychological relevance: schizotypy and subclinical depression. These constructs are typically positioned at the border between normative variations and clinical pathology, making them ideal candidates for examining how curiosity may function, or malfunction, under different psychological conditions. A dimensional approach in non-clinical samples further enables the study of curiosity-related processes across the full spectrum of individual differences while minimizing confounds associated with clinically significant psychopathology.

Despite its everyday familiarity, curiosity remains a complex and multidimensional construct. At a rather basic level, it can be distinguished into a stable dispositional trait (i.e., trait curiosity) and a momentary, situational state (i.e., state curiosity), both of which interact and influence how individuals engage with novelty and uncertainty^2,3^. Berlyne^4^, who focused on trait curiosity, further distinguished between perceptual curiosity, triggered by novel sensory stimuli, and epistemic curiosity (EC), which describes a drive to acquire new knowledge. More recent frameworks, such as the Five-Dimensional Curiosity Scale Revised (5DCR)^5,6^, offer a more nuanced view, identifying (six) different dimensions, including Joyous Exploration, Deprivation Sensitivity, Stress Tolerance, Thrill Seeking, and Social Curiosity (General and Covert). Notably, these dimensions are not only of theoretical importance since each has been linked to specific motivational, emotional, and cognitive processes. For example, Joyous Exploration refers to the intrinsic enjoyment of discovering new information, while Deprivation Sensitivity is marked by a more aversive urge to resolve information gaps. The latter may be particularly relevant in contexts of psychological discomfort or compulsive ideation, both of which are common in schizotypal traits.

Schizotypy is a multifaceted personality trait characterized by cognitive-perceptual distortions, interpersonal difficulties, and disorganized thought patterns. It has often been examined through a risk perspective for schizophrenia-spectrum disorders but, important for our approach, it is increasingly recognized as a dimensional trait that is present in varying degrees across the general population^7–10^. Therefore, specific schizotypy profiles may be beneficial, while others could have detrimental effects^11^. For instance, individuals with high levels of unusual experiences, but without accompanying negative or disorganized traits, reported psychological well-being comparable to average and low-schizotypy profiles^12^. This is compatible with the observation that individuals high in positive schizotypy (i.e., unusual perceptual experiences and magical ideation) but low in negative schizotypy (i.e., reduced anhedonia and interpersonal deficits) show elevated resilience and well-being, despite markedly reduced self-concept clarity, reflecting a less clearly defined and internally consistent sense of self^11^. Interestingly, curiosity correlated with different scales of the Wisconsin Schizotypy Scale, including both positive (e.g., magical ideation) and negative (e.g., physical anhedonia) symptoms^13^, and in another rare study, an overlap in perceptual processing between creativity and curiosity, but not schizotypy, was found^14^. Given the scarcity of studies directly examining these associations, further systematic investigation is needed to clarify the nature and direction of these relationships.

Subclinical depression, on the other hand, is characterized by reduced motivation, lowered energy levels, and a diminished sense of pleasure in everyday activities^15,16^. While it does not meet the full diagnostic criteria for major depressive disorder, subclinical depression can significantly impair daily functioning and is a known risk factor for a more severe psychopathology^15^. From a motivational point of view, depression is often linked to anhedonia and reduced behavioral activation, factors likely to suppress curiosity, particularly its more active or exploratory dimensions. Empirical research has begun to support this notion, by showing that higher trait curiosity is associated with lower levels of depressive symptoms, potentially through enhanced subjective well-being^17^. In a longitudinal study, trait curiosity in children was negatively associated with depression in adulthood, suggesting that childhood curiosity may serve as a protective factor against depression, possibly by fostering greater confidence in the future^18^. Moreover, individuals with greater fluctuations in daily curiosity experience higher depressive symptoms and lower life satisfaction^19^, indicating that not only the absolute level but also stability of curiosity appears to be relevant with regard to mental health. Finally, experimentally induced negative affect was associated with less state curiosity^20^, further suggesting that negative mood states can dampen the motivational drive to seek new information and engage with novel stimuli – core components of state EC.

Depression, schizotypy and curiosity appear to share neurobiological substrates within the dopaminergic mesolimbic system^2,21,22^. For instance, patients with psychosis show abnormal salience processing in the dopaminergic midbrain^23^, and individuals at ultra-high risk for psychosis exhibit altered activation and connectivity within a hippocampal, basal ganglia, midbrain circuit during salience processing^24^. Being in a state of high curiosity, that can be induced by unknown answers to trivia questions^25,26^ or magic tricks^27,28^, leads to enhanced mesolimbic activity and promotes subsequent memory formation. Patients with depression, on the other hand, typically show reduced mesolimbic activity, including impaired reward-prediction-error signaling in the striatum and reduced striatal-midbrain connectivity^29,30^. These neurobiological parallels further suggest that heightened curiosity in certain domains could coexist with changes in mental well-being.

Taken together, there is evidence that both schizotypy and subclinical depression might correlate with changes in specific facets of curiosity. Based on previous research revealing positive longitudinal effects of trait curiosity^17^, as well as evidence that negative affect can influence momentary curiosity states^19^, we therefore explored whether subclinical depressive symptoms may be negatively related to both trait and state curiosity. With the evidence for links between curiosity and schizotypy being mixed^13,14^, we hypothesize that associations between schizotypy and curiosity vary across trait dimensions. More specifically, and following the differentiation between positive and negative symptoms in schizophrenia, we expect positive associations between schizotypy and exploratory curiosity dimensions (i.e., Joyous Exploration, Thrill Seeking) as well as heightened Deprivation Sensitivity, but negative associations with Stress Tolerance.

To test these hypotheses, we used validated self-report measures of the multidimensional structure of trait curiosity (5DCR) together with a previously employed measure of state EC^31^ in a sample of healthy adults varying in schizotypal traits (experiment 1, *n* = 238) and subclinical depressive symptoms (experiment 2, *n* = 264). By adopting the 5DCR framework, we aimed to test for overall associations and to explore which specific facets of curiosity (e.g., Joyous Exploration, Stress Tolerance, or Social Curiosity) are most sensitive to variations in schizotypal traits (as measured with the LSHS and ESI) and subclinical depressive symptoms (as measured with the PHQ9), see methods. This approach aligns with growing calls for dimensional, transdiagnostic perspectives in psychological research^32^, viewing schizotypy and depression not as binary categories but as continua relevant to a broad spectrum of mental functioning. Understanding how curiosity is diminished in subclinical depression and altered in schizotypy may reveal early motivational dysfunctions and non-pathological cognitive divergences that impact on functioning in distinct ways. Therefore, our overall goal was to clarify the role of curiosity within subclinical populations and explore its potential as a protective factor or early marker of psychological vulnerability.

## Methods

### Experiment 1 – schizotypy and curiosity

#### Participants

298 healthy humans participated in experiment 1, an online experiment using SoSci Survey^33^. Primary inclusion criteria encompassed a minimum age of 18 years and no history of participation in previous online studies using our state EC paradigm, which excluded 12 subjects from further analyses. On a secondary level, 32 participants were excluded based on invalid catch-trials (see below) and data from 16 subjects were classified as outliers, with their scores on either the curiosity or schizotypy measurement falling more than three standard deviations above or below the group mean. In total, data from 238 subjects (age range 18–81 years, M = 31.03, SD = 13.6) were analyzed. The study was conducted entirely in German (including instructions, questionnaires, and stimuli), and sufficient German language proficiency was therefore required for participation.

#### Study design and procedure

##### Trait curiosity

To assess trait curiosity, we used the German version of the Five Dimensions of Curiosity Scale Revised (5DCR)^34^. The questionnaire measures the following six dimensions: Joyous Exploration, Deprivation Sensitivity, Stress Tolerance, Social Curiosity General, Social Curiosity Covert, and Thrill Seeking. Joyous Exploration captures the tendency to seek out new information and experiences with enthusiasm and excitement; Deprivation Sensitivity reflects a drive to resolve gaps in knowledge that cause discomfort or frustration; Stress Tolerance assesses the ability to cope with uncertainty, complexity, and novel situations without feeling overwhelmed; Social Curiosity General measures the general interest in gaining knowledge about how other people think, feel, and behave; Social Curiosity Covert captures a more discreet, often indirect, form of interest in others’ private lives; Thrill Seeking reflects the willingness to seek out exciting, novel, and sometimes risky experiences. Four items per dimension are rated on a 7-point Likert-scale, and higher values indicate greater expression of the respective trait curiosity dimension.

##### State curiosity

Following our previous experiments^31^ subjects were presented with a series of trivia questions from various categories, including “science and nature,” “history,” and “entertainment”. The questions were taken from the board game “Trivial Pursuit – Classic Edition,” (German version, 2017). After each question, they rated their confidence in knowing the answer on a Likert scale from 1 “I am very confident I don’t know the answer” to 6: “I am very confident I know the answer”. Subsequently, they indicated their level of curiosity to learn the answer, from 1: “I am not very curious to know the answer” to 6: “I am very curious to know the answer”. Questions which were confidently rated as known (i.e., 6 on the knowledge dimension) were disregarded, and subjects were presented with a replacement item. Four catch-trials were embedded in this part of the questionnaire, instructing participants to select a number between one and six to ensure the questions were read and answered attentively. The average curiosity values for unknown questions were used as a marker of state EC. The answers to all trivia questions were displayed at the end of the study.

##### Schizotypy

The short version of the Eppendorf Schizophrenia Inventory (ESI) was administered to quantify schizotypal traits and psychosis proneness in non-clinical populations. It includes four subscales: Attention and Speech Impairment scale, Deviant Perception scale, Ideas of Reference scale, and Auditory Uncertainty scale. Attention and Speech Impairment captures difficulties in maintaining focus and coherent speech, Deviant Perception measures unusual perceptual experiences and distortions in sensory processing, Ideas of Reference reflects the tendency to interpret neutral events as personally significant, and Auditory Uncertainty assesses experiences of ambiguous or distorted auditory perceptions. All items were answered on a four-point Likert scale, ranging from 0 “not true at all” to 3 “exactly true”^35^. Higher values indicate greater levels of schizotypal experiences across the respective dimension. The ESI has been positively evaluated for assessing schizotypal aspects of personality in non-clinical settings^36^.

Additionally, we used the Launay-Slade Hallucination Scale (LSHS)^37^ to assess auditory hallucinations, consisting of twelve items to be answered on a five-point Likert scale from 0 “certainly does not apply to me” to 4 “certainly applies to me”. Higher values indicate a greater tendency toward hallucinatory experiences. The scale was originally developed to study hallucination-like experiences on a continuum with psychotic symptoms, therefore allowing us to focus on aspects of schizotypy mirroring the positive symptoms present in schizophrenia. It is not diagnostic, but captures subclinical experiences that may be relevant in schizotypy, dissociation, and general proneness to psychosis-like phenomena. However, both measures do not explicitly tap into anhedonia, or other negative symptoms of schizotypy.

##### Sociodemographic information

All subjects were asked for their age, gender, highest educational degree (according to the German educational system), employment status and current residency (country and state). Highest educational degree included: no degree, main school (“Hauptschule”), secondary school (“Realschule”), apprenticeship (“abgeschlossene Ausbildung”), advanced technical college entrance qualification (“Fachabitur”), German high school diploma (“Abitur”), degree from an institution of higher education (“including University and University of Applied Sciences”), and still pupil at school.

#### Data analysis

Data were preprocessed using Matlab^38^ and further analyzed with jamovi^39^ and R^40^. For trait curiosity, mean values for each dimension were computed. Stress Tolerance was re-coded to align with the direction of the other dimensions, such that higher values indicate higher trait Stress Tolerance. For state EC, individual mean values were computed, with 21 scorings per subject. As for schizotypy, sum scores were computed for the LSHS and for the four ESI subscales. Internal consistency was generally adequate across trait and schizotypy scales (Cronbach’s α ranged from 0.732 to 0.835) and excellent for our state curiosity instrument (Cronbach’s α = 0.921), which is comparable to other studies^41,42^.

We computed a correlation matrix for all relevant variables. The matrix included the six dimensions of trait curiosity, state EC, both schizotypy measures (LSHS, ESI subscales), and age. We applied Holm-Bonferroni correction for multiple comparisons to the family-wise alpha level of 0.05. For each set of *m* = 78 correlations, p-values were ordered in ascending order and compared sequentially to adjusted alpha levels of 0.05 / (*m* – *i* + 1). The procedure was stopped once a p-value exceeded its adjusted threshold, and all subsequent correlations were treated as non-significant.

### Experiment 2 – subclinical depression and curiosity

#### Participants

324 healthy humans participated in experiment 2, an online experiment using SoSci Survey^33^. Based on the primary inclusion criteria as specified for experiment 1, we excluded 12 subjects from further analyses due to previous participation. 21 participants were excluded based on invalid catch-trials. For the curiosity measures, an outlier analysis revealed nine subjects with values falling more than three standard deviations above or below the group mean. Additionally, we applied a cutoff of 14 for the PHQ9, as values ≥15 indicate moderate to severe depressive symptoms. This excluded 22 subjects from the analysis. In total, data from 264 subjects (age range 18–83 years, *M* = 24.7, *SD* = 7.95) were analyzed. The study was conducted entirely in German (including instructions, questionnaires, and stimuli), and sufficient German language proficiency was therefore required for participation.

#### Study design and procedure

The study design was identical to experiment 1 with the exception of the clinical questionnaire: Instead of ESI and LSHS, subjects were presented with the Patient Health Questionnaire (PHQ9)^43^. It is a widely used self-report measure based on DSM-IV criteria. Participants rated the frequency of nine depressive symptoms over the past two weeks on a 4-point Likert scale from 0 “not at all” to 3 “nearly every day”, yielding a total score ranging from 0 to 27. Higher scores indicate greater severity of depressive symptoms.

#### Data analysis

Data were preprocessed using Matlab^38^ and further analyzed using jamovi^39^ and R^40^. Preprocessing was identical to experiment 1. Internal consistency of the trait curiosity questionnaires was comparable to experiment 1 (Cronbach’s α ranged from 0.688 to 0.838) and again excellent for our state curiosity instrument (Cronbach’s α = 0.916). Reliability was acceptable for the PHQ9 (Cronbach’s α = 0.763), slightly lower than expected for non-clinical populations^44^.

A correlation matrix for all relevant variables was computed. The matrix included the six dimensions of trait curiosity, state EC, the PHQ9 sum score, and age. We applied Holm-Bonferroni correction for multiple comparisons to the family-wise alpha level of 0.05. as described for experiment 1. We included *m* = 36 correlations, p-values were ordered in ascending order and compared sequentially to adjusted alpha levels of 0.05/(*m* – *i* + 1) until a p-value exceeded its adjusted threshold. All subsequent correlations were treated as non-significant.

### Ethics approval, consent and pre-registration

This study (experiment 1 and 2) was approved by the local ethics committee of the University of Luebeck, Germany (Ethikkommission der Universität zu Lübeck). All participants gave written informed consent, in accordance with the Declaration of Helsinki, before taking part in the study. This study was not pre-registered.

## Results

### Experiment 1 – schizotypy and curiosity

Detailed results can be found in Table S1. For all statistics, we only report effects that survived Holm-Bonferroni correction.

For the six trait curiosity dimensions, we observed the following correlation patterns: Joyous Exploration positively correlated with Stress Tolerance (*r* = .359), Social Curiosity General (*r* = .461) and Thrill Seeking (*r* = .264; all *p* < .001). Further, there was a significant positive intercorrelation between Social Curiosity General and Social Curiosity Covert (*r* = .341, *p* < .001). In contrast, higher Deprivation Sensitivity was associated with lower Stress Tolerance (*r* = − .278, *p* < .001). Except for Social Curiosity General (*r* = .216, *p* < .001), none of the trait curiosity dimensions were significantly correlated with state EC.

**Fig. 1:**
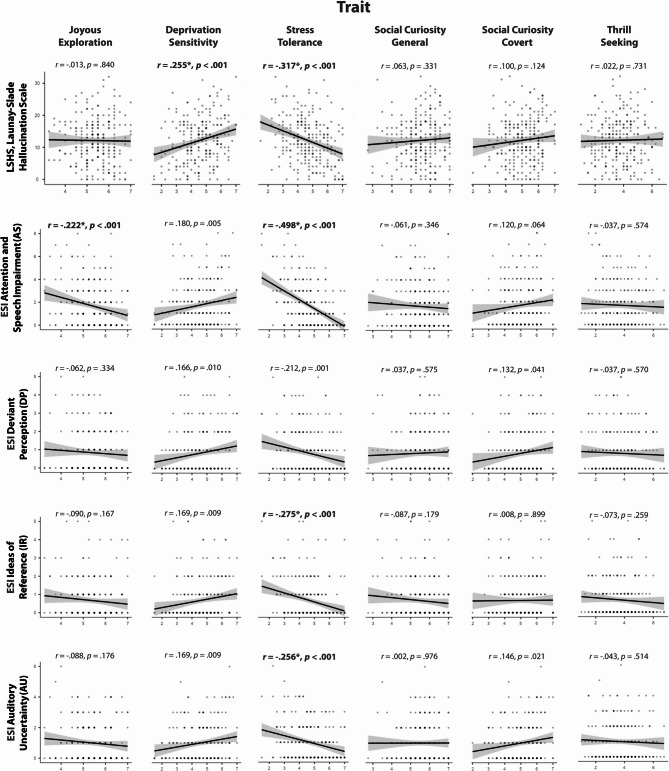
Correlations between trait curiosity dimensions and markers of schizotypy (experiment 1). Significant correlations emerged between Joyous Exploration and ESI Attention and Speech (negative) as well as Deprivation Sensitivity and LSHS (positive). Stress Tolerance was negatively correlated with all schizotypy measures except ESI Deviant Perception. Data are presented as correlation coefficients (Pearson’s r) and uncorrected p-values. Abbreviations: ESI – Eppendorf Schizophrenia Inventory; AS – Attention and Speech Impairment scale; DP – Deviation Perception; IR – Ideas of Reference; AU – Auditory Uncertainty; LSHS – Launay-Slade Hallucination Scale.

For trait curiosity and schizotypy measures, there was a significant negative correlation between Joyous Exploration and ESI Attention and Speech Impairment (*r* = − .222, *p* < .001). Additionally, Deprivation Sensitivity positively correlated with the LSHS (*r* = .254, *p* < .001). Stress Tolerance showed a negative correlation with the LSHS (*r* = − .314, *p* < .001), ESI Attention and Speech Impairment (*r* = − .498, *p* < .001), ESI Ideas of Reference (*r* = − .274, *p* > .001) as well as ESI Auditory Uncertainty (*r* = − .259, *p* < .001). Both Social Curiosity General and Social Curiosity Covert, and Thrill Seeking were not significantly correlated with any of the schizotypy measures (all p > corrected alpha). All correlations between trait curiosity and schizotypy can be seen in Fig. 1 and Table S1.

Age significantly correlated negatively with Social Curiosity Covert (*r* = − .307, *p* < .001) and Thrill Seeking (*r* = − .221, *p* < .001).

Taken together, Joyous Exploration, Deprivation Sensitivity and Stress Tolerance significantly correlated with specific aspects of schizotypy, while age was only associated with Social Curiosity Covert and Thrill Seeking. State EC, on the other hand, did not significantly correlate with any other measure of investigation except Social Curiosity General. For details, including intercorrelations between schizotypy scales and Bayes factors, see Table S1.

### Experiment 2 – subclinical depression and curiosity

Detailed results can be found in Table S2. Similar to Experiment 2, we only report effects that survived Holm-Bonferroni correction. As expected, we again observed several positive intercorrelations for trait curiosity. Most prominently, Joyous Exploration significantly correlated with all trait dimensions, with correlation coefficients ranking between *r* = .215 and 0.470. Thrill Seeking significantly correlated with Stress Tolerance (*r* = .298, *p* < .001). Lastly, both social trait curiosity dimensions (General and Covert) were positively correlated (*r* = .465, *p* < .001).

For subclinical depression and trait curiosity, both Joyous Exploration (*r* = − .257, *p* = .001) and Stress Tolerance (*r* = − . 262, *p* < .001) showed a positive correlation with PHQ9. All other measures of trait curiosity did not significantly correlate with PHQ9.

**Fig. 2:**
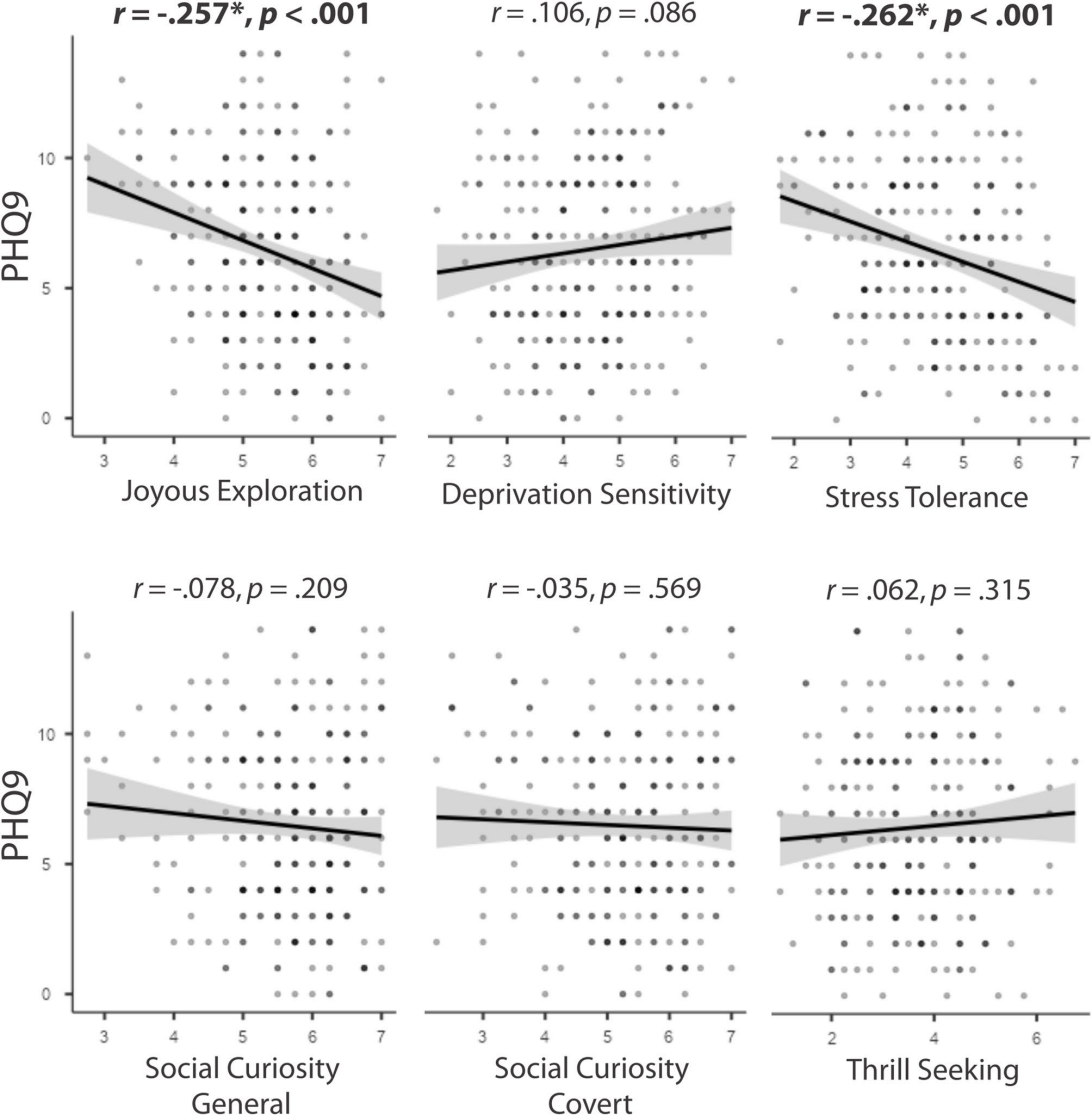
Correlations between subclinical depression (PHQ9) and trait curiosity dimensions (experiment 2). Subclinical depression significantly negatively correlated with Joyous Exploration and Stress Tolerance. Data are presented as correlation coefficients (Pearson’s r) and uncorrected p-values. Abbreviations: PHQ9 = Patient Health Questionnaire-9.

In contrast to experiment 1, state EC showed a significant positive correlation with Joyous Exploration (*r* = .467, *p* < .001), Social Curiosity General (*r* = .303, *p* < .001), and Social Curiosity Covert (*r* = .274, *p* < .001), but not trait Deprivation Sensitivity, Stress Tolerance or Thrill Seeking. All correlations between trait curiosity and subclinical depression (PHQ9) can be seen in Fig. 2 and Table S2.

Comparable with experiment 1, Social Curiosity Covert (*r* = − .215, *p* < .001) and Thrill Seeking (*r* = − .208, *p* = .001) showed a negative relationship with age.

Taken together, PHQ9 scores were negatively associated with Joyous Exploration and Stress Tolerance, while no significant relationship emerged with state EC. State EC was however associated with several trait curiosity dimensions. Again, age correlated with Social Curiosity Covert and Thrill Seeking. For details, including Bayes factors, see Table S2.

## Discussion

Understanding how state and trait curiosity relate to schizotypy and subclinical depression may offer novel insights into early motivational dysfunctions and non-pathological cognitive variation relevant to psychological functioning. Therefore, we tested two human samples varying in schizotypal traits (experiment 1, *n* = 238) and subclinical depressive symptoms (experiment 2, *n* = 264) with measures of trait curiosity and state epistemic curiosity (EC). Our results show that both schizotypal characteristics (experiment 1) and subclinical depressive symptoms (experiment 2) were associated with lower Joyous Exploration and Stress Tolerance. In addition, Deprivation Sensitivity was positively associated with schizotypal characteristics. State EC, on the other hand, did not significantly correlate with schizotypy or depressive symptoms, and across both experiments, Thrill Seeking and Social Curiosity Covert decreased with age. This pattern suggests partially overlapping associations of schizotypy and subclinical depression with lower exploratory and uncertainty-tolerant aspects of trait curiosity, while higher Deprivation Sensitivity showed a more selective association with schizotypal characteristics. Therefore, these findings highlight the importance of distinguishing between specific aspects of curiosity when examining mental health, as they may reflect both protective and risk-related motivational profiles.

With regard to schizotypy (experiment 1), the correlation analysis provided evidence for selective associations with specific dimensions of trait curiosity. Most consistently, lower Stress Tolerance was linked to several schizotypy-related measures, including impaired attention and speech (ESI Attention and Speech), delusions of reference (ESI Ideas of Reference) and hallucinatory experiences (ESI Auditory Uncertainty, LSHS), suggesting that individuals who experience greater discomfort when facing uncertainty, ambiguity, or novelty may also be more prone to psychosis-like experiences. In addition, lower Joyous Exploration was associated with more attentional and speech-related impairments (ESI Attention and Speech), whereas lower Deprivation Sensitivity related to fewer or less pronounced hallucinatory tendencies (LSHS). Conversely, higher Stress Tolerance and Joyous Exploration may reflect a greater capacity to engage with uncertainty and novelty in an adaptive manner, potentially buffering against schizotypy-related cognitive and perceptual experiences^11,45^. Together, these findings point toward a differential relationship between schizotypy and trait curiosity, in which reduced tolerance for uncertainty and heightened aversive responses to information gaps appear particularly relevant. This pattern is partly compatible with previous work linking schizotypy to both positive and negative aspects of curiosity^13^, while also extending these findings by showing that specific curiosity dimensions may relate differently to distinct schizotypal characteristics.

In experiment 2, subclinical depressive symptoms were negatively associated with Joyous Exploration and Stress Tolerance. These findings are in line with previous work linking higher trait curiosity to lower levels of depression and greater subjective well-being^17^, and further support the notion that specific curiosity dimensions may act as protective factors against affective vulnerability across the lifespan^18,19^. More specifically, individuals characterized by greater intrinsic enjoyment of acquiring new knowledge (Joyous Exploration) and a higher tolerance for uncertainty and ambiguity (Stress Tolerance) showed lower levels of subclinical depressive symptoms. Interestingly, social curiosity, particularly Social Curiosity General, was not significantly associated with depressive symptoms. Given that social withdrawal is a common feature of depression, this absence of an association may indicate a dissociation between self-reported interest in social information and actual social behavior. However, as social withdrawal is not directly assessed by the PHQ9, future studies using more specific measures of social functioning are needed. Taken together, this pattern suggests that subclinical depression may not reflect a general reduction in curiosity, but rather specific reductions in joyous exploration and lower tolerance of uncertainty.

With regard to state EC, our correlation analyses (experiment 2) indicated positive associations with several dimensions of trait curiosity, particularly Joyous Exploration and both dimensions of Social Curiosity (General and Covert). This pattern is consistent with previous research suggesting that individuals reporting higher levels of trait curiosity also tend to experience more frequent curiosity-related states in everyday life^46,47^. Interestingly, these associations were limited to curiosity dimensions characterized by positive engagement with novel information and social exploration, whereas dimensions more closely related to the regulation of uncertainty or discomfort, such as Stress Tolerance, showed no significant relationship with state EC. Notably, these associations emerged only in experiment 2, but not in experiment 1, which could be explained by at least three factors. First, the two experiments were conducted in independent samples and therefore likely differed in uncontrolled sample characteristics. Second, experiment 1 included substantially more questionnaire items due to the extended assessment of schizotypal characteristics, potentially increasing fatigue or reducing attentional engagement during the state EC task. Third, the larger number of variables included in experiment 1 resulted in a more conservative correction for multiple comparisons, which may have reduced statistical sensitivity for detecting weaker state–trait associations. Together, these considerations suggest that the absence of significant state–trait correlations in experiment 1 should be interpreted cautiously rather than as evidence against a relationship between trait and state curiosity.

Despite these associations between state EC and several trait curiosity dimensions, state EC itself did not significantly correlate with either schizotypal characteristics or subclinical depressive symptoms. This absence of an association is noteworthy given previous work linking specific schizotypy profiles to cognitive exploration and well-being^11,12^, as well as studies showing that experimentally induced negative affect can reduce momentary curiosity states^20^. One possible interpretation is that schizotypy and subclinical depression may relate less to the dynamic experience of momentary epistemic curiosity, and more strongly to stable dispositional aspects of curiosity, particularly those linked to positive exploratory motivation and the ability to tolerate uncertainty and ambiguity. In this context, the observed associations with Joyous Exploration and Stress Tolerance may reflect shared mechanisms related to motivational engagement with novelty and uncertainty.

This interpretation is also interesting in light of previous neurobiological frameworks proposing overlapping dopaminergic and mesolimbic mechanisms across curiosity, schizotypy, and depression^2,21,22,29^. While curiosity states have repeatedly been associated with mesolimbic engagement during information seeking^25–28^, depression and psychosis-proneness are often linked to altered salience processing and dysregulated reward-related signaling^23,24,29,30^. However, our findings suggest that these overlaps may be more closely related to enduring motivational dispositions than to the momentary expression of state EC in non-clinical populations. Future studies combining behavioral assessments with neuroimaging approaches, such as fMRI, and potentially psychopharmacological manipulations, may help to clarify whether shared neural mechanisms contribute differently to state and trait expressions of curiosity across clinical and non-clinical populations.

Trait curiosity is hypothesized to decrease with age^48,49^, but very few studies have investigated the developmental changes of different facets of trait curiosity. In both of our experiments, Thrill Seeking and Social Curiosity Covert were negatively related to age, which helps to close this gap. The decline in Thrill Seeking is consistent with existing personality research on sensation seeking and related constructs^50–52^, and aligns with observations that the propensity for risky behavior decreases with age^53,54^. The negative correlation between age and Social Curiosity Covert matches previous findings on age-related reductions in interpersonal curiosity^55^ and further indicates that older subjects take less interest in discreetly gathering information about others. However, curiosity dimensions that were more consistently related to mental health in the present study (i.e., Joyous Exploration, Deprivation Sensitivity, and Stress Tolerance) were not associated with age. This is partly incompatible with previous work^55^ and suggests that age-related changes in curiosity may be more selective and dimension-specific than previously assumed. Therefore, future research should further investigate how multidimensional curiosity profiles change across the lifespan to better understand their relevance for psychological and cognitive well-being^55^.

Finally, we would like to point out the following strengths and limitations. First, our online experiments are based on cross-sectional designs, which is a time and cost-efficient approach, but individual changes and developmental trajectories can only be inferred. Second, we used a state of the art multi-facet curiosity questionnaire^6^ in combination with sub-clinical measures. The latter allowed us to identify early psychological markers and risk factors before clinical thresholds are reached, offering insights into intervention targets. However, our findings and conclusions may not fully generalize to clinically diagnosed populations, limiting their direct applicability to clinical treatment contexts. Third, although negative schizotypy commonly encompasses social or introverted anhedonia, the instruments used here primarily captured cognitive-perceptual and hallucinatory aspects. Therefore, future studies may benefit from employing measures that more explicitly assess anhedonic components, which could reveal distinct or overlapping mediating pathways. Fourth, our rationale and theoretical frameworks include the assumption of underlying neurobiological principles. This, however, can only be investigated using brain imaging techniques, which could be fruitful methods in future research.

To conclude, our findings underscore the complex interplay between trait curiosity, schizotypal characteristics and subclinical depression. Across both experiments, lower Joyous Exploration and Stress Tolerance were consistently associated with less favorable mental health profiles, whereas heightened Deprivation Sensitivity appeared to be more specifically linked to schizotypal characteristics. These findings suggest that different dimensions of curiosity may reflect distinct motivational and affective processes, with some potentially acting as protective factors and others relating to psychological vulnerability. Together, our results highlight the value of a multidimensional perspective on curiosity for understanding individual differences in subclinical mental health and motivational functioning.

## Supplementary Information

Below is the link to the electronic supplementary material.

Supplementary Material 1

## Data Availability

All data is available at The Open Science Framework (OSF, https://osf.io/x3sm9).
