# Supplementary material for: Schizotypy and subclinical depression relate to specific trait curiosity dimensions
Source: Sci Rep. 2026 Jul 17;16:22672. doi: 10.1038/s41598-026-61554-y (PMC13385751; doi:10.1038/s41598-026-61554-y)
Supplement: Supplementary file 1 — Supplementary Material 1 [file 41598_2026_61554_MOESM1_ESM.docx]

**Supplementary material**

**Schizotypy and subclinical depression relate to specific trait curiosity dimensions**

Heike Sönnichsen^1^, Alexandra Sobczak^1^, Tineke Steiger^1^, Nico Bunzeck^1, 2*^

^1^ Department of Psychology, University of Luebeck, 23562 Luebeck, Germany

^2^ Center of Brain, Behavior and Metabolism (CBBM), University of Luebeck, Luebeck, Germany

*Corresponding authors:

Heike Sönnichsen

Department of Psychology, University of Luebeck, Maria-Goeppert-Strasse 9a, 23562 Luebeck, Germany

Phone: +49-(0) 451 3101 3603

E-Mail: he.soennichsen@uni-luebeck.de

Nico Bunzeck

Department of Psychology, University of Luebeck, Maria-Goeppert-Strasse 9a, 23562 Luebeck, Germany

Phone: +49-(0) 451 3101 3600

E-Mail: nico.bunzeck@uni-luebeck.de

## **Table S1.** Correlation matrix Experiment 1with LSHS and ESI within the range of +/- 3SD. Listed mean values (M) and standard deviations (SD), correlation coefficients (Pearson’s r), p-values, and Bayes factors (BF10). Abbreviations: EC: Epistemic Curiosity, LSHS: Launay-Slade Hallucination Scale, ESI: Eppendorf Schizophrenia Inventory, AS: Attention and Speech Impairment, DP: Perceptual Deviation, IR: Ideas of Reference, AU: Auditory Uncertainty.

|  | | **M (SD)** | | **Joyous**  **Exploration** | | | | | **Deprivation**  **Sensitivity** | | **Stress**  **Tolerance** | | | **Social Curiosity**  **General** | | | **Social Curiosity**  **Covert** | | | **Thrill**  **Seeking** | | | **State**  **EC** | | | **LSHS** | | | **ESI AS** | | | **ESI DP** | | | **ESI IR** | | | **ESI AU** | | **age** | | |  |
| --- | --- | --- | --- | --- | --- | --- | --- | --- | --- | --- | --- | --- | --- | --- | --- | --- | --- | --- | --- | --- | --- | --- | --- | --- | --- | --- | --- | --- | --- | --- | --- | --- | --- | --- | --- | --- | --- | --- | --- | --- | --- | --- | --- |
| Joyous  Exploration |  | | 5.41 (0.79) | |  | — |  |  | |  | |  |  | |  |  | |  |  | |  |  | |  |  | |  |  | |  |  | |  |  |  |  |  | |  | |  |  |  |
|  |  | |  | |  | — |  |  | |  | |  |  | |  |  | |  |  | |  |  | |  |  | |  |  | |  |  | |  |  |  |  |  | |  | |  |  |  |
| Deprivation  Sensitivity |  | | 4.54 (1.21) | |  | .208 |  | — | |  | |  |  | |  |  | |  |  | |  |  | |  |  | |  |  | |  |  | |  |  |  |  |  | |  | |  |  |  |
|  |  | |  | |  | .001 |  | — | |  | |  |  | |  |  | |  |  | |  |  | |  |  | |  |  | |  |  | |  |  |  |  |  | |  | |  |  |  |
|  |  | |  | |  | 14.622 |  | — | |  | |  |  | |  |  | |  |  | |  |  | |  |  | |  |  | |  |  | |  |  |  |  |  | |  | |  |  |  |
| Stress  Tolerance |  | | 4.70 (1.21) | |  | .359 | * | -.279 | | * | | — |  | |  |  | |  |  | |  |  | |  |  | |  |  | |  |  | |  |  |  |  |  | |  | |  |  |  |
|  |  | |  | |  | < .001 |  | < .001 | |  | | — |  | |  |  | |  |  | |  |  | |  |  | |  |  | |  |  | |  |  |  |  |  | |  | |  |  |  |
|  |  | |  | |  | **8.15e5** |  | **1.05e3** | |  | | — |  | |  |  | |  |  | |  |  | |  |  | |  |  | |  |  | |  |  |  |  |  | |  | |  |  |  |
| Social Curiosity  General |  | | 5.50 (1.21) | |  | .461 | * | .187 | |  | | .050 |  | | — |  | |  |  | |  |  | |  |  | |  |  | |  |  | |  |  |  |  |  | |  | |  |  |  |
|  |  | |  | |  | < .001 |  | .004 | |  | | .444 |  | | — |  | |  |  | |  |  | |  |  | |  |  | |  |  | |  |  |  |  |  | |  | |  |  |  |
|  |  | |  | |  | **1.09e11** |  | 5.219 | |  | | 0.109 |  | | — |  | |  |  | |  |  | |  |  | |  |  | |  |  | |  |  |  |  |  | |  | |  |  |  |
| Social  Curiosity  Covert |  | | 5.50 (0.89) | |  | .041 |  | .080 | |  | | -.187 |  | | .340 | * | | — |  | |  |  | |  |  | |  |  | |  |  | |  |  |  |  |  | |  | |  |  |  |
|  |  | |  | |  | .526 |  | .220 | |  | | .004 |  | | < .001 |  | | — |  | |  |  | |  |  | |  |  | |  |  | |  |  |  |  |  | |  | |  |  |  |
|  |  | |  | |  | 0.099 |  | 0.171 | |  | | 5.308 |  | | **1.42e5** |  | | — |  | |  |  | |  |  | |  |  | |  |  | |  |  |  |  |  | |  | |  |  |  |
| Thrill  Seeking |  | | 3.72 (1.22) | |  | .264 | * | .059 | |  | | .172 |  | | .187 |  | | .137 |  | | — |  | |  |  | |  |  | |  |  | |  |  |  |  |  | |  | |  |  |  |
|  |  | |  | |  | < .001 |  | .367 | |  | | .008 |  | | .004 |  | | .034 |  | | — |  | |  |  | |  |  | |  |  | |  |  |  |  |  | |  | |  |  |  |
|  |  | |  | |  | **379.344** |  | 0.122 | |  | | 2.775 |  | | 5.121 |  | | 0.755 |  | | — |  | |  |  | |  |  | |  |  | |  |  |  |  |  | |  | |  |  |  |
| State EC |  | | 3.43 (1.08) | |  | .166 |  | -.019 | |  | | -.021 |  | | .216 | * | | .077 |  | | -.073 |  | | — |  | |  |  | |  |  | |  |  |  |  |  | |  | |  |  |  |
|  |  | |  | |  | .010 |  | .768 | |  | | .745 |  | | < .001 |  | | .238 |  | | .259 |  | | — |  | |  |  | |  |  | |  |  |  |  |  | |  | |  |  |  |
|  |  | |  | |  | 2.118 |  | 0.085 | |  | | 0.086 |  | | **21.807** |  | | 0.162 |  | | 0.153 |  | | — |  | |  |  | |  |  | |  |  |  |  |  | |  | |  |  |  |
| LSHS |  | | 12.07 (6.89) | |  | -.013 |  | .255 | | * | | -.317 | * | | .063 |  | | .100 |  | | .022 |  | | .081 |  | | — |  | |  |  | |  |  |  |  |  | |  | |  |  |  |
|  |  | |  | |  | .840 |  | < .001 | |  | | < .001 |  | | .331 |  | | .124 |  | | .731 |  | | .211 |  | | — |  | |  |  | |  |  |  |  |  | |  | |  |  |  |
|  |  | |  | |  | .083 |  | **208.967** | |  | | **1.95e4** |  | | 0.130 |  | | 0.262 |  | | 0.086 |  | | 0.177 |  | | — |  | |  |  | |  |  |  |  |  | |  | |  |  |  |
| ESI AS |  | | 1.67 (1.84) | |  | -.222 | * | .180 | |  | | -.498 | * | | -.061 |  | | .120 |  | | -.037 |  | | -.012 |  | | .307 | * | | — |  | |  |  |  |  |  | |  | |  |  |  |
|  |  | |  | |  | < .001 |  | .005 | |  | | < .001 |  | | .346 |  | | .064 |  | | .573 |  | | .850 |  | | < .001 |  | | — |  | |  |  |  |  |  | |  | |  |  |  |
|  |  | |  | |  | **30.002** |  | 3.804 | |  | | **2.67e13** |  | | 0.126 |  | | 0.446 |  | | 0.095 |  | | 0.083 |  | | **8.74e3** |  | | — |  | |  |  |  |  |  | |  | |  |  |  |
| ESI DP |  | | 0.84 (1.17) | |  | -.062 |  | .166 | |  | | -.212 |  | | .037 |  | | .132 |  | | -.037 |  | | .096 |  | | .366 | * | | .309 | * | | — |  |  |  |  | |  | |  |  |  |
|  |  | |  | |  | .344 |  | .010 | |  | | .001 |  | | .575 |  | | .041 |  | | .570 |  | | .139 |  | | < .001 |  | | < .001 |  | | — |  |  |  |  | |  | |  |  |  |
|  |  | |  | |  | 0.127 |  | 2.158 | |  | | **17.315** |  | | 0.095 |  | | 0.643 |  | | 0.095 |  | | 0.241 |  | | **1.65e6** |  | | **1.00e4** |  | | — |  |  |  |  | |  | |  |  |  |
| ESI IR |  | | 0.67 (1.09) | |  | -.090 |  | .169 | |  | | -.275 | * | | -.087 |  | | .008 |  | | -.073 |  | | .122 |  | | .320 | * | | .394 | * | | .337 | * | — |  |  | |  | |  |  |  |
|  |  | |  | |  | .167 |  | .009 | |  | | < .001 |  | | .179 |  | | .899 |  | | .259 |  | | .060 |  | | < .001 |  | | < .001 |  | | < .001 |  | — |  |  | |  | |  |  |  |
|  |  | |  | |  | 0.209 |  | 2.446 | |  | | **785.247** |  | | 0.199 |  | | 0.082 |  | | 0.153 |  | | 0.472 |  | | **2.43e4** |  | | **2.90e7** |  | | **1.08e5** |  | — |  |  | |  | |  |  |  |
| ESI AU |  | | 1.00 (1.22) | |  | -.088 |  | .169 | |  | | -.256 | * | | .002 |  | | .149 |  | | -.043 |  | | .125 |  | | .447 | * | | .424 | * | | .382 | * | .471 | * | — | |  | |  |  |  |
|  |  | |  | |  | .176 |  | .009 | |  | | < .001 |  | | .986 |  | | .021 |  | | .514 |  | | .054 |  | | < .001 |  | | < .001 |  | | < .001 |  | < .001 |  | — | |  | |  |  |  |
|  |  | |  | |  | 0.202 |  | 2.413 | |  | | **225.474** |  | | 0.081 |  | | 1.131 |  | | 0.100 |  | | 0.516 |  | | **1.71e10** |  | | **9.67e9** |  | | **8.23e6** |  | **4.36e11** |  | — | |  | |  |  |  |
| age |  | | 31.06 (13.58) | |  | .012 |  | -.061 | |  | | .105 |  | | -.154 |  | | -.307 | * | | -.217 |  | | .082 |  | | -.204 |  | | -.069 |  | | -.106 |  | .014 |  | -.066 | |  | | — |  |  |
|  |  | |  | |  | .856 |  | .352 | |  | | .107 |  | | .017 |  | | < .001 |  | | < .001 |  | | .207 |  | | .002 |  | | .291 |  | | .103 |  | .831 |  | .310 | |  | | — |  |  |
|  |  | |  | |  | 0.082 |  | 0.125 | |  | | 0.296 |  | | 1.343 |  | | **8.88e3** |  | | **23.087** |  | | 0.179 |  | | **11.453** |  | | 0.141 |  | | 0.304 |  | 0.083 |  | 0.135 | |  | | — |  |  |

Note. N=238. * p < adj. alpha (Holm-Bonferroni). Bold values indicate BF10 > 30 (very strong evidence).

## **Table S2**. Correlation matrix Experiment 1 with PHQ < 15. Listed mean values (M) and standard deviations (SD), correlation coefficients (Pearson’s r), p-values, and Bayes factors (BF10). Abbreviations: EC: Epistemic Curiosity, PHQ9: Patient Health Questionnaire.

| Correlation Matrix | | | | | | | | | | | | | | | | | | | | | | |
| --- | --- | --- | --- | --- | --- | --- | --- | --- | --- | --- | --- | --- | --- | --- | --- | --- | --- | --- | --- | --- | --- | --- |
|  | | **M (SD)** | | **Joyous**  **Exploration** | | **Deprivation**  **Sensitivity** | | **Stress**  **Tolerance** | | **Social**  **Curiosity**  **General** | | **Social**  **Curiosity**  **Covert** | | **Thrill**  **Seeking** | | **State EC** | | **PHQ9** | | **age** | | |
| Joyous  Exploration |  | 5.33 (0.82) |  | — |  |  |  |  |  |  |  |  |  |  |  |  |  |  |  | |  |  |
|  |  |  |  | — |  |  |  |  |  |  |  |  |  |  |  |  |  |  |  | |  |  |
|  |  |  |  | — |  |  |  |  |  |  |  |  |  |  |  |  |  |  |  | |  |  |
| Deprivation  Sensitivity |  | 4.43 (1.11) |  | 0.258 | * | — |  |  |  |  |  |  |  |  |  |  |  |  |  | |  |  |
|  |  |  |  | < .001 |  | — |  |  |  |  |  |  |  |  |  |  |  |  |  | |  |  |
|  |  |  |  | **578.916** |  | — |  |  |  |  |  |  |  |  |  |  |  |  |  | |  |  |
| Stress  Tolerance |  | 4.48 (1.16) |  | 0.315 | * | -0.094 |  | — |  |  |  |  |  |  |  |  |  |  |  | |  |  |
|  |  |  |  | < .001 |  | 0.129 |  | — |  |  |  |  |  |  |  |  |  |  |  | |  |  |
|  |  |  |  | **5.97e4** |  | 0.243 |  | — |  |  |  |  |  |  |  |  |  |  |  | |  |  |
| Social  Curiosity  General |  | 5.64 (0.92) |  | 0.470 | * | 0.136 |  | 0.070 |  | — |  |  |  |  |  |  |  |  |  | |  |  |
|  |  |  |  | < .001 |  | 0.027 |  | 0.260 |  | — |  |  |  |  |  |  |  |  |  | |  |  |
|  |  |  |  | **9.45e12** |  | 0.865 |  | 0.145 |  | — |  |  |  |  |  |  |  |  |  | |  |  |
| Social  Curiosity  Covert |  | 5.25 (1.12) |  | 0.215 | * | 0.034 |  | -0.100 |  | 0.465 | * | — |  |  |  |  |  |  |  | |  |  |
|  |  |  |  | < .001 |  | 0.578 |  | 0.105 |  | < .001 |  | — |  |  |  |  |  |  |  | |  |  |
|  |  |  |  | **35.199** |  | 0.090 |  | 0.284 |  | **4.12e12** |  | — |  |  |  |  |  |  |  | |  |  |
| Thrill  Seeking |  | 3.37 (1.18) |  | 0.228 | * | 0.163 |  | 0.298 | * | 0.167 |  | 0.078 |  | — |  |  |  |  |  | |  |  |
|  |  |  |  | < .001 |  | 0.008 |  | < .001 |  | 0.006 |  | 0.209 |  | — |  |  |  |  |  | |  |  |
|  |  |  |  | **79.497** |  | 2.535 |  | **1.43e4** |  | 3.080 |  | 0.169 |  | — |  |  |  |  |  | |  |  |
| State EC |  | 3.47 (1.04) |  | 0.467 | * | 0.165 |  | -0.047 |  | 0.303 | * | 0.274 | * | 0.070 |  | — |  |  |  | |  |  |
|  |  |  |  | < .001 |  | 0.007 |  | 0.446 |  | < .001 |  | < .001 |  | 0.257 |  | — |  |  |  | |  |  |
|  |  |  |  | **5.72e12** |  | 2.753 |  | 0.103 |  | **2.03e4** |  | **1.97e3** |  | 0.146 |  | — |  |  |  | |  |  |
| PHQ9 |  | 6.47 (3.41) |  | -0.257 | * | 0.106 |  | -0.262 | * | -0.078 |  | -0.035 |  | 0.062 |  | -0.110 |  | — |  | |  |  |
|  |  |  |  | < .001 |  | 0.086 |  | < .001 |  | 0.209 |  | 0.569 |  | 0.315 |  | 0.073 |  | — |  | |  |  |
|  |  |  |  | **574.073** |  | 0.335 |  | **811.831** |  | 0.169 |  | 0.091 |  | 0.127 |  | 0.379 |  | — |  | |  |  |
| age |  | 24.71 (7.95) |  | 0.000 |  | -0.014 |  | 0.051 |  | -0.128 |  | -0.215 | * | -0.208 | * | 0.050 |  | 0.006 |  | | — |  |
|  |  |  |  | 0.999 |  | 0.820 |  | 0.410 |  | 0.038 |  | < .001 |  | < .001 |  | 0.415 |  | 0.924 |  | | — |  |
|  |  |  |  | 0.077 |  | 0.079 |  | 0.108 |  | 0.653 |  | **35.824** |  | 24.449 |  | 0.107 |  | 0.077 |  | | — |  |
| Note. N=264. * p < adj. alpha (Holm-Bonferroni). Bold values indicate BF10 > 30 (very strong evidence). | | | | | | | | | | | | | | | | | | | | | | |
